# Multimorbidity in Patients with Chronic Liver Disease: A Population-Based Study in the EpiChron Cohort, Spain

**DOI:** 10.3390/jcm13237198

**Published:** 2024-11-27

**Authors:** Aída Moreno-Juste, Beatriz Poblador-Plou, Clara Laguna-Berna, Belén Cruces-Mateo, Elisa Lenotti, Alejandro Santos-Mejías, Luis A. Gimeno-Feliú, Antonio Gimeno-Miguel

**Affiliations:** 1 EpiChron Research Group, Aragon Health Sciences Institute (IACS), IIS Aragón, Miguel Servet University Hospital, 50009 Zaragoza, Spain; bpoblador.iacs@aragon.es (B.P.-P.); clagunab.iacs@aragon.es (C.L.-B.); bcruces@iisaragon.es (B.C.-M.); asantosm@ext.aragon.es (A.S.-M.); lugifel@gmail.com (L.A.G.-F.); agimenomi.iacs@aragon.es (A.G.-M.); 2 Illueca Primary Care Health Centre, Aragon Health Service (SALUD), 50250 Illueca, Spain; 3 Network for Research on Chronicity, Primary Care, and Health Promotion (RICAPPS), Institute of Health Carlos III (ISCIII), 28029 Madrid, Spain; 4 Department of Clinical and Experimental Sciences, University of Brescia, 25123 Brescia, Italy; e.lenotti@unibs.it; 5 San Pablo Primary Care Health Centre, Aragon Health Service (SALUD), 50003 Zaragoza, Spain; 6 Department of Medicine, Dermatology and Psychiatry, University of Zaragoza, 50009 Zaragoza, Spain

**Keywords:** chronic liver disease, multimorbidity, comorbidity, population-based study

## Abstract

**Background/Objectives**: Chronic liver disease (CLD) is highly relevant in clinical practice due to its increasing incidence and associated mortality. We aimed to exhaustively characterize the multimorbidity of patients with CLD. **Methods**: This is a retrospective observational study of patients aged 18 years and older with a diagnosis of CLD in 2015 in the EpiChron Cohort (Spain). We analyzed the prevalence of comorbidities according to sex and age, conducted a logistic regression analysis with CLD as the independent variable and each of the comorbidities as dependent variables to obtain odds ratios (OR) adjusted for age and sex, and performed an exploratory factor analysis to identify the presence of multimorbidity patterns. **Results**: A total of 6836 adults had a diagnosis of CLD (mean age 61.6 years; 62.5% women), with an average of 13 co-existing chronic conditions. Arterial hypertension, lipid metabolism disorders, diabetes, obesity, and musculoskeletal diseases were the most frequent diseases. From the list of 110 chronic conditions analyzed, 75 were systematically associated with CLD, including (OR, 95% confidence interval) chronic pancreatitis (41.2, 33.5–50.6) and inherited metabolic disorders (14.9, 11.8–18.8). Three multimorbidity patterns were identified in both men and women, including cardiovascular, metabolic-geriatric, and mental-substance use, with some differences by sex. **Conclusions**: Multimorbidity is the norm in patients with CLD. Our results reveal that a comprehensive and integral approach based on person-centered care, which should take into account their entire spectrum of multimorbidity, is necessary during the care of this type of patient in clinical practice.

## 1. Introduction

Chronic liver disease (CLD) is a common clinical condition consisting of a progressive deterioration of liver function related to a continuous process of inflammation, destruction, and regeneration of the liver parenchyma [1]. This process can be due to a wide spectrum of etiologies, including prolonged excessive alcohol consumption (alcoholic liver disease), metabolic alterations (non-alcoholic fatty liver disease), infections (chronic viral hepatitis), autoimmune diseases, genetic disorders, and hepatotoxic drugs, among others [2,3]. This severe damage causes the liver to lose its ability to repair itself, beginning with hepatosteatosis, which leads to fibrosis, cirrhosis, and, ultimately, the appearance of hepatocellular carcinoma [1,2].

Representing the fifth cause of death in the European Union, CLD is also responsible for high rates of disability and intensive use of healthcare services [3,4]. Its prevalence has experienced an increasing trend in recent decades. Approximately 1.5 billion people suffer from CLD in the world [4,5], and its average prevalence in Europe is 0.83% [6]. According to data from the Global Burden of Disease Study, the age-standardized incidence rate of this disease was 20.7 cases per 100,000 inhabitants in 2015, which represents an increase of 13% since 2000 [4]. The epidemiology of CLD has undergone a change in recent years, reflecting, on the one hand, the implementation of large-scale hepatitis B vaccination and hepatitis C treatment programs and, on the other hand, the increasing prevalence of metabolic syndrome and excessive alcohol consumption [3].

The initial symptoms of CLD may initially be nonspecific, including fatigue, anorexia, and weight loss, or may manifest clinically with the development of complications such as esophageal varices, ascites, jaundice, hepatic encephalopathy, hepatorenal syndrome, hepatopulmonary syndrome, and coagulopathies [2,7]. Once diagnosed, the goal of CLD treatment is to stop disease progression and complications, which requires a multidisciplinary approach to correct the underlying cause, control portal hypertension, and treat individual signs of the disease. This requires a holistic and integrated approach by an interprofessional team including primary care physicians, specialists in gastroenterology, hepatology and nephrology, liver transplant teams if necessary, nutritionists to provide advice on diet, social workers, and community nurses [2].

The spectrum of symptoms and conditions of patients with CLD is not limited to those caused by the disease itself. There may be many other coexisting diseases related to other pathophysiological mechanisms, both concordant and discordant. The coexistence of two or more chronic diseases in the same individual is known as multimorbidity [8]. Multimorbidity is currently the norm rather than the exception in clinical practices around the world, and it can result in interactions between diseases and between the drugs used to treat them, complicating the clinical management of chronic patients [9].

The comorbidity of CLD has already been studied in the literature; however, most studies have focused on describing the specific comorbidities of some of the etiological diseases of CLD, such as alcoholic liver disease [10] and nonalcoholic fatty liver [11], or focus on its complications, such as ascites [12] and hepatocellular carcinoma [13]. Predictors of CLD have also been reported, including hypertension, insulin resistance, diabetes mellitus, and obesity. Most previous studies on the comorbidity of CLD were based on the calculation of disease prevalence rates or on regression models to calculate the likelihood of the appearance of specific comorbidities based on the presence of CLD. No studies, except for one, have analyzed the existence of multimorbidity patterns in patients with CLD; however, that study only focused on patients with hepatocellular carcinoma [13]. To the best of our knowledge, there is no study that has analyzed the complete multimorbidity spectrum of CLD as a whole.

A comprehensive analysis of multimorbidity in patients with CLD could help to identify the most frequent and associated coexisting diseases, providing us with useful information for their early identification, diagnosis, and even prevention, whereas the analysis of their multimorbidity patterns could help to identify the profiles of patients susceptible to differentiated clinical management based on their specific pattern of coexisting diseases.

Our population study based on real-life data aimed to comprehensively analyze the comorbidity of CLD by means of the characterization of the most prevalent chronic comorbidities in these patients, the identification of those comorbidities systematically associated with CLD regardless of their prevalence, and the identification and clinical description of the existing multimorbidity patterns in patients with CLD.

## 2. Materials and Methods

### 2.1. Study Design and Population

We conducted a retrospective analytical observational study in the EpiChron Cohort, which integrates pseudonymized demographic and clinical information of all of the users of the public health system of Aragón (Spain), who represent approximately 95% of its reference population (1.3 million inhabitants). The baseline characteristics of the EpiChron Cohort can be found elsewhere [14]. The conformation of this cohort for research on the epidemiology of multimorbidity and chronic diseases was favorably evaluated by the Research Ethics Committee of the Community of Aragón (CEICA; approval code PI17/0024). Given the epidemiological nature of this study, which used anonymized data that were presented only at an aggregate level, the obligation to obtain informed consent from the patients was waived by CEICA.

For this study, we selected, as reference population, all of the people aged 18 years and older from the cohort who were registered as users of the public health system in 2015 and who were also registered during the previous year to have at least one year of complete information for their demographic and clinical characterization. Of these, we selected all of the patients with a diagnosis of CLD recorded in their electronic health records for the study of their multimorbidity.

### 2.2. Variables and Data Sources

For all of the patients included in this study, we analyzed their sex and age as of 1 January 2015 and all of their chronic comorbidities registered in both primary and hospital care.

The diagnoses included in the electronic health records were originally coded using the International Classification of Primary Care, 1st edition (ICPC-1), in the case of primary care, and the International Classification of Diseases, 9th edition, Clinical Modification (ICD-9-CM), in the case of hospital care. These codes were grouped into broader mutually exclusive diagnostic categories called Expanded Diagnostic Clusters (EDCs) based on their clinical and diagnostic similarities using the Johns Hopkins Adjusted Clinical Groups (ACG^®^) (version 11.0, The Johns Hopkins University, Baltimore, MD, USA) [15]. The purpose of using this software was to transform the thousands of potential different codes into a more manageable list of 264 diagnoses in order to avoid duplicate diagnoses and facilitate the counting of diseases. From this list, only the 114 EDCs proposed as chronic in the study by Salisbury et al. [16] were taken into account. Chronic diseases were defined as those diseases that typically last six months or more, including past conditions that require current care, conditions with a high risk of recurrence, or past conditions that have ongoing implications for patient management. We grouped some diagnoses so that a total of 110 conditions were finally analyzed. A list of diseases used is presented as Supplementary Material (Table S1). Subjects with CLD were identified as those having the EDC code “GAS05: Chronic liver disease,” which included all cases of CLD regardless of their type or etiology.

All of the information was gathered from patients’ electronic medical history of primary care, hospital discharge reports (through the Minimum Basic Data Set; CMBD), and clinical-administrative databases (user database, BDU).

### 2.3. Statistical Analysis

For the comprehensive analysis of the multimorbidity of patients with CLD, we conducted a three-phase analysis. First, we carried out a descriptive analysis of the demographic and clinical characteristics of the study population. For the identification of the most frequent comorbidities, we analyzed the prevalence of each comorbidity in the total population and stratified by sex and age (i.e., 18–44, 45–65, and >65 years), which was presented as frequencies and percentages.

Secondly, for the identification of those comorbidities systematically associated with CLD, we used logistic regression models to calculate the risk of occurrence of each comorbidity (dependent variable) based on the presence or absence of CLD (independent variable). As a result, we obtained odds ratios (ORs) accompanied by their 95% confidence intervals (95% CI), which were calculated as unadjusted crude ORs and as age- and sex-adjusted ORs. For the comparison of adjusted ORs, we used the Bonferroni correction method for multiple comparisons (for a total of 85 disease comparisons with at least five cases in both men and women), establishing statistical significance at *p* < 0.00059.

Finally, for the analysis of the existence of associations among diseases in the form of multimorbidity patterns in patients with CLD, we carried out an exploratory factor analysis. This technique allowed us to reduce the number of explanatory variables in the data set to a smaller number of latent variables, which can be interpreted as groups of variables (in our case diseases) that share a common underlying causal factor. This analysis was carried out in each sex and age group. To increase the clinical relevance of the results and facilitate their interpretation, we only included in the analysis those comorbidities with a prevalence greater than 5%. Each comorbidity was represented in the analysis as a binary variable indicating presence (1) or absence (0), which allowed for the construction of a tetrachoric correlation matrix. To extract the factors, we used the main factor method, and to facilitate its interpretation, we applied an oblique rotation of the factors (Oblimin). To choose the number of factors (i.e., patterns) to extract, we used scree plots of the eigenvalues from the correlation matrix ordered in descending order. This visualization allowed us to identify the inflection point, which indicates the optimal number of factors to extract. This statistical criterion was accompanied by the clinical assessment of the different results.

In each pattern, we included those chronic diseases with a loading factor or factor score (a value that ranges between −1 and 1, representing the strength of association of each diagnosis within each pattern) equal to or greater than 0.30, allowing each disease to be in more than one pattern. To determine the degree of suitability of the sample to use for factor analysis, we performed the Kaiser–Meyer–Olkin (KMO) test; moreover, the value of this parameter varies between 0 and 1, with values closer to 1 representing a greater goodness of fit.

Once the patterns in each subpopulation were obtained, and to facilitate their clinical interpretation, we named the patterns based on the composition of diseases and the names already described in the bibliography with the help of two clinical care specialists in family and community medicine from the research group.

All statistical analyzes were carried out in Stata (Version 12.0, StataCorp LLC, College Station, TX, USA) and R (Version 3.6.3, The R Foundation for Statistical Computing, Vienna, Austria).

## 3. Results

Of the 954,168 people aged 18 years or older in the EpiChron Cohort in 2015 (505,313 women and 448,855 men; mean age of 52.3 years), 6836 people had an active diagnosis of CLD in their electronic medical record (mean age of 61.6 years; 62.5% women; Table 1). These data translate into a prevalence of CLD in adults of 0.51% in women and 0.95% in men, with 95% of cases accumulating in people over 40 years of age. Multimorbidity was present in 99% of people with CLD, who showed a high burden of comorbidity, with an average of 13 chronic conditions.

### 3.1. Most Prevalent Chronic Conditions in Patients with CLD

Some cardiometabolic diseases, such as arterial hypertension, lipid metabolism disorders, diabetes, obesity, and musculoskeletal diseases like arthritis, were among the most frequent diseases found in the population with CLD (Table 1). These conditions were also some of the most prevalent in the overall cohort. Although these diseases had similar behavior in the different population groups studied, there were some relevant differences, such as the high prevalence of disorders related to substance use in men from an early age, as well as depression and cancer (Table 2).

### 3.2. Chronic Conditions Associated with the Presence of CLD

From the original list of 110 conditions analyzed, 75 were associated with CLD, with their prevalence being higher than expected (Table 3). The most systematically associated comorbidity was chronic pancreatitis (adjusted OR 41.2), followed by inherited metabolic disorders and hemophilia.

### 3.3. Multimorbidity Patterns in Patients with CLD

Regarding the exploratory factor analysis, unacceptable values below 0.50 were obtained for the KMO parameter in the different age and sex strata, indicating that these samples were not suitable for conducting an analysis of this type [17]. Moreover, the Heywood phenomenon occurred in some cases, which is sometimes related to the existence of collinearity between variables [18]. Consequently, we carried out the analyses stratified only by sex, so that multimorbidity patterns were determined in men and women, but not in the different age intervals. When the analyses were carried out stratified by sex, this type of phenomenon did not occur, and acceptable sample adequacy indices were obtained (KMO of 0.76 in men and 0.65 in women).

**Figure 1:**
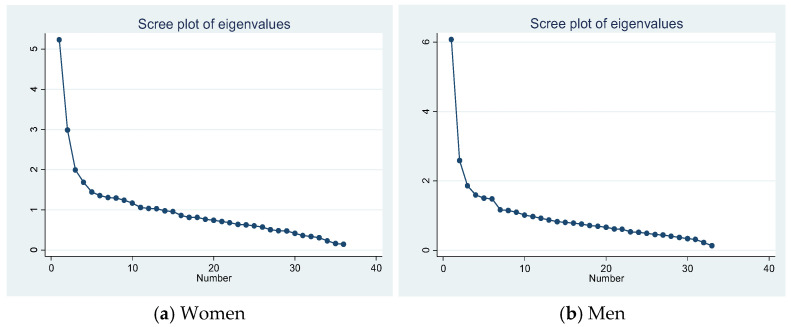
Scree plots used to determine the optimal number of factors (i.e., multimorbidity patterns) to extract in women and men with chronic liver disease. The inflection point of the curve indicates the approximate optimal number of factors to extract, a decision that was complemented by clinical judgment.

To choose the number of patterns to extract in each analysis, we based our decision on the sedimentation graphs presented in Figure 1, in which the inflection point of the eigenvalues occurred between three and six factors in both sexes, but not in a clear way. Therefore, this criterion was complemented by the assessment of two clinicians of the team, who chose the optimal number of patterns based on the clinical interpretation of the different possible alternatives, always looking for the model with greater parsimony that explained the greatest degree of variance with the minimum number of variables.

**Figure 2:**
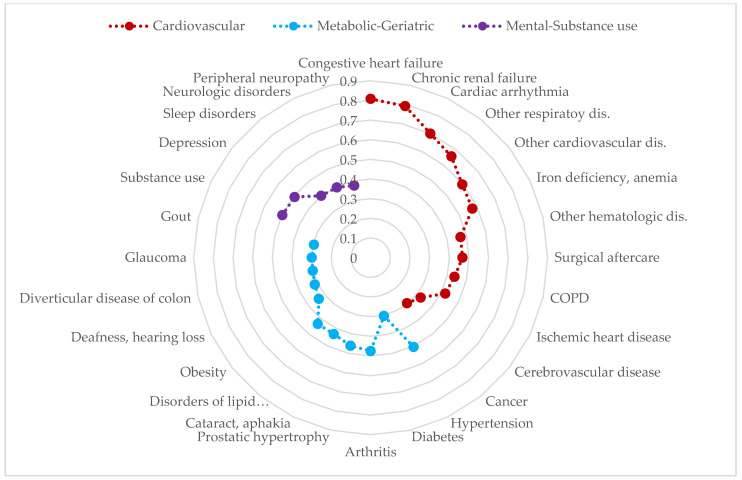
Multimorbidity patterns in adult men with chronic liver disease in the EpiChron Cohort. COPD: Chronic obstructive pulmonary disease. Dis.: disorders. Disorders of lipid…: Disorders of lipid metabolism. The Y axis represents the loading factor (between 0 and 1) of each disease within its respective pattern. Only those diseases with loading factors ≥ 0.30 are represented.

**Figure 3:**
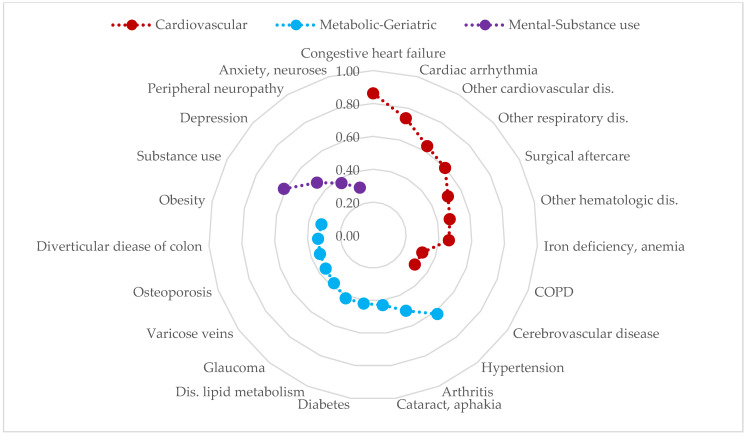
Multimorbidity patterns in adult women with chronic liver disease in the EpiChron Cohort. COPD: Chronic obstructive pulmonary disease. Dis.: disorders. The Y axis represents the loading factor (between 0 and 1) of each disease within its respective pattern. Only those diseases with loading factors ≥ 0.30 are represented.

Finally, we decided to extract three factors in each sex, resulting in three patterns of multimorbidity in both men (Figure 2) and women (Figure 3). These patterns were named cardiovascular, metabolic-geriatric, and mental-substance use in both men and women, although they showed slight differences in their composition depending on sex.

The cardiovascular pattern included cardiovascular diseases such as congestive heart failure, arrhythmia, anemia, and ischemic disease, as well as chronic obstructive pulmonary disease (COPD), other respiratory disorders, and cerebrovascular disease. In the case of men, cancer was also added to this pattern.

The metabolic-geriatric pattern presented with chronic metabolic conditions such as diabetes, obesity, and lipid metabolism disorder (in men, also gout), which were joined by conditions that seem more typical of advanced age like glaucoma, deafness, cataracts, and arthritis (and also osteoporosis in women).

The mental-substance use pattern was composed of substance use, depression, sleep disorders, and neuritis (and also anxiety in women).

## 4. Discussion

In this study, we observed that multimorbidity is the norm rather than the exception in patients with CLD, which is frequently accompanied by other diseases, like arterial hypertension, lipid metabolism disorders, diabetes, obesity, and musculoskeletal diseases, with some relevant differences between the sexes, such as the higher prevalence in men of disorders related to substance use from an early age, as well as of depression and cancer. This multimorbidity seems to cluster in three different multimorbidity patterns (cardiovascular, metabolic-geriatric, and mental-substance use) in both men and women, although with slight differences in their composition depending on sex.

The prevalence rates of CLD obtained in our study are similar to and consistent with the average prevalence of CLD described in the European Union, which is around 0.83% [6], and with the fact that excessive alcohol consumption, one of the most common etiologies of CLD, is more common in men than in women [19].

The prevalence rate of multimorbidity is higher than those observed in previous articles reporting multimorbidity rates of 80% and a lower number of comorbidities [11,20], which is probably due to the fact that our study exhaustively analyzed all possible chronic conditions, and it was not based on a limited number of them. It has been demonstrated that the risk and severity of non-alcoholic liver disease increase with the number of components of metabolic syndrome present, and obesity is considered the biggest risk factor for it [11]. Similar results were observed in people with non-alcoholic liver disease in Russia, of whom almost 80% had at least two metabolic comorbidities, with the most common ones being overweight/obesity (81%), hypercholesterolemia (75%), and type 2 diabetes (17%) [11]. In a population-based study in Sweden, high blood pressure was more prevalent (33%) than type 2 diabetes (29%) and obesity (24%). In the Manitoba Follow-up Study, hypertension, insulin resistance/diabetes mellitus, and obesity were reported as the greatest predictors of the appearance of CLD [21]. However, the majority of studies focused on describing the specific comorbidities of some of the etiological diseases of CLD, such as alcoholic liver disease [10] and nonalcoholic fatty liver [11], or on its complications, such as ascites [12] and hepatocellular carcinoma [13]. Among the most prevalent comorbidities, immunological conditions such as allergic rhinitis and arthritis were observed. Allergic rhinitis was found to be one of the most prevalent comorbidities in both men and women aged between 18 and 39 years. However, arthritis was much more prevalent in women aged 40 years and older compared with their male counterparts. This difference could be due to the fact that osteoarthritis is well known to be more prevalent in women than in men. Furthermore, women used to present worse osteoarthritis outcomes, partially due to the experience of more pain symptoms despite having similar levels of structural damage. This has been related to the different innate immune system activation between genders, including an increase in macrophages and a decrease in monocytes in women, or the different expression of proinflammatory cytokines between the sexes, among others [22].

Surprisingly, 75 conditions of the original list of 110 were associated with CLD in our study, and their prevalence was higher than expected. This could be due to the multifactorial etiology of CLD and to the fact that the liver represents an important organ with many functions [2], whose alteration and damage could, in turn, be the cause of many other chronic disorders. In fact, among the conditions that showed a higher degree of association with CLD, we observed conditions that are both possible causes (e.g., inherited metabolic disorders, chronic viral infection, substance use, autoimmune diseases, diabetes, and obesity [2,3]) and potential consequences (e.g., coagulation disorders, thrombosis, gastroesophageal reflux, and chronic renal/respiratory failure) of CLD [2,23]. The fact that the diseases associated the most with CLD were inherited liver diseases, which are a group of metabolic and genetic defects like Wilson disease, alpha-1 antitrypsin deficiency, or hereditary hemochromatosis, might be explained by the fact that these conditions typically cause early chronic liver disease [24]. The association between chronic pancreatitis and CLD could be related to the fact that both diseases are major alcohol-related diseases in most countries. These conditions are developmentally related and share a number of functional similarities, such the common features of alcohol-induced injury, although the severity of these diseases does not depend on whether they manifest at the same time or not [25]. The liver is involved in the synthesis of coagulation factor proteins and blood coagulation inhibitors and in the activation of coagulation products, among others. Consequently, it would be expected that patients with CLD are more prone to suffer from coagulation disorders [26].

Although the relationship between CLD and comorbidities has been studied, there are not many studies that analyze the association of these comorbidities in the form of patterns. In addition, we have observed the presence of three patterns, and Mu et al. identified three main comorbidity patterns in patients with hepatocellular carcinoma, which included the following: (1) cirrhosis, hepatitis B, portal hypertension, and ascites; (2) hypertension, diabetes mellitus, coronary heart disease, and cerebral infarction; and (3) hypoproteinemia, electrolyte disorders, gastrointestinal bleeding, and hemorrhagic anemia [13].

Of the three patterns analyzed, the cardiovascular pattern has been widely described in the literature and is one of the most consistent in the general population [27,28]. Although it has not been specifically described in patients with CLD, it is one of the main cause of hospital admissions [12] and death in patients with non-alcoholic fatty liver disease [29,30]. In Germany, it was observed that among hospital admissions of CLD patients, the diagnosis of respiratory diseases with infection had the highest mortality rate of 21.6%, followed by cerebrovascular disease with a rate of 15.5% [12]. The development of these cardiovascular complications is related to obesity [12,29]. It is thought that the hyperdynamic circulation in cirrhosis provides some protection against overt heart failure, atherosclerosis, and ischemic events, but peripheral arterial disease, acute myocardial infarction, and heart failure were predictors of mortality in the CirCom study [23].

The metabolic-geriatric pattern is also one of the most consistently described in the general population [27], and, in the specific case of CLD, it makes sense that there is a profile of patients with this type of multimorbidity, since metabolic syndrome and fatty liver disease are the most common causes of CLD [11,31]. We observed that this pattern was mainly composed of obesity, diabetes, and lipid metabolism disorder (due to low HDL and high triglycerides), which are considered metabolic risk factors of CLD, and diabetes is a predictor of severe outcomes [31]. Non-alcoholic fatty liver disease is recognized as the hepatic manifestation of the metabolic syndrome that represents a cluster of metabolic abnormalities, such as hyperlipidemia, glucose intolerance, obesity, and systemic hypertension [11], comorbidities that we found to be associated with CLD. The fact that this pattern is associated with geriatric or aging diseases could be due to the fact that older CLD patients are precisely those who have had this metabolic etiology, and not the one related to the excessive consumption of alcohol and related factors that may cause these patients to have a lower life expectancy [32]. However, due to the technique used (factor analysis), which groups diseases and not people, it is not possible to know the average age of the patients of this multimorbidity pattern to corroborate this hypothesis.

The mental-substance use pattern was composed of substance use, depression, sleep disorders, and neuritis and has been previously described in the literature, but mainly in young and adult men, while, in women, a mental pattern with anxiety and depression is usually observed, but not associated with substance use [27,28]. As in the previous case, this pattern of multimorbidity makes clinical sense, since it would represent another of the most frequent etiologies of CLD, being alcoholic liver disease, due to excessive alcohol consumption [10,23]. Knowing that this pattern is also associated with problems such as depression and sleep disorders is important in order to characterize the specific needs of these patients [33] and proactively seek their appearance and diagnosis in order to prevent worsening health outcomes. In many cases, depression is associated with stigmatization in patients with liver disease, and it is associated with a lack of social support and a decrease in the tendency to seek health care [33]. In the development of the CirCom score, Jepsen et al. considered that mental disease could be a predictor of mortality, due to its association with substance abuse and suicide risk; however, they observed that schizophrenia was indeed an adverse prognostic factor, but depression and bipolar disorder were not associated with mortality [23]. They did not find other studies that have examined the prognostic impact of psychiatric diseases in cirrhosis [23].

The negatives outcomes of CLD patients are not only due to their comorbidities and clinical complexity, but also to biological (i.e., ageing, frailty, multimorbidity, mental disease, dependency, and malnutrition) and non-biological (i.e., socioeconomic, behavioral, environmental, and cultural) variables [34]. Lifestyle factors, including smoking, alcohol consumption, physical inactivity, adiposity [30], and poor diet [29], play a key role in the incidence of CLD, as well as their complications. That is the reason why the promotion of lifestyle interventions among CLD patients in the early stage of disease course is necessary, in order to prevent cardiovascular risk factors [29], transitions to metabolic complications, and death [30]. Also, social factors like limitations in daily living due to their disease, loneliness, low income, stigmatization, and isolation play an important role in the evolution of the disease decreasing the quality of life of these patients [33]. Patients have highlighted the need for information to understand and manage CLD and awareness and support from healthcare professionals to better cope with the disease [33]. An interprofessional team that provides a holistic and integrated approach is needed for a CLD patient to achieve the best possible outcomes. The earlier signs and symptoms of a complication are identified, the better the prognosis and outcome [2].

One of the most important limitations of our study is related to its cross-sectional design, which did not allow for the establishment of causal relationships between the existence of CLD and the comorbidities analyzed, nor to study the order of appearance or time of progression of the diseases within each multimorbidity pattern. On the other hand, given the multifactorial etiology of CLD, it would have been interesting to differentiate between the type of each case of CLD according to its etiology (e.g., alcoholism, non-alcoholic fatty liver, or viral infection). Unfortunately, this information was not available in our cohort due to the codification of diseases according to the disease grouping software used, which grouped all cases of CLD under the EDC ‘Chronic liver disease,’ regardless of their etiology. Furthermore, the stage of chronic liver disease was not analyzed, and the comorbidities associated with CLD could be different depending on the stage of the disease. In this sense, we encourage the conduct of future studies that take into account the type and stage of CLD in order to ascertain whether they influence the conformation and composition of multimorbidity patterns. In addition, it was not possible to analyze other variables of interest for the interpretation of the results, such as the socioeconomic and educational level of the patients, genetic variables, or information on lifestyles (e.g., physical activity and the consumption of alcohol and tobacco), which were not available in the EpiChron Cohort. It is also worth noting that the factor analysis presented limitations for the study of the different sex and age strata, and the analysis had to be performed stratified only by sex.

One of the main strengths of this study lies in its population-based nature, since all cases of CLD in the reference population were analyzed. Furthermore, we exhaustively analyzed the comorbidity of CLD based on the analysis of virtually all chronic diseases (and not only the most relevant or frequent ones) contained in the clinical history and recorded by a professional (and not self-referred by the patients or from surveys).

## 5. Conclusions

Multimorbidity is the norm in patients with CLD, who have a huge disease burden. Our study revealed that, although the majority of the most prevalent diseases in these patients are also among the most prevalent in the general population, there are other diseases that, although much less prevalent, are systematically associated with CLD, such as chronic pancreatitis, coagulation disorders, and inherited metabolic disorders. Our study also revealed the existence of three differentiated multimorbidity patterns in women and men with CLD, named as cardiovascular, metabolic-geriatric, and mental-substance use. These results, which should be validated in other contexts, indicate that the care of these patients in clinical practice should follow an approach that takes into account all of their comorbidities in a global and comprehensive manner, trying to avoid a follow-up based only on the recommendations of clinical practice guidelines for specific individual diseases, in order to provide them with person-centered care.

## Figures and Tables

**Table 1:** Demographic and clinical characteristics of the adult population with chronic liver disease in the EpiChron Cohort (Aragon, Spain).

Sex (*n*, %)	Women (2563; 37.5%)	Men (4273; 62.5%)	Total (6836; 100%)
Age (*n*, %)			
18–39 years	142 (5.54)	236 (5.52)	378 (5.53)
40–64 years	1170 (45.7)	2375 (55.6)	3545 (51.9)
≥65 years	1251 (48.8)	1662 (38.9)	2913 (42.6)
Mean age (s.e ^1^), years	63.3 (0.29)	60.6 (0.21)	61.6 (0.17)
Multimorbidity ^2^ (%)	99.6	99.1	99.3
Number of chronic diseases, mean (s.d. ^3^)	14.1 (7.31)	12.5 (7.21)	13.1 (7.29)
Most prevalent chronic diseases (EDC ^4^, %)	Arterial hypertension (49.9)	Arterial hypertension (49.9)	Arterial hypertension (48.0)
	Disorders of lipid metabolism (42.3)	Disorders of lipid metabolism (41.3)	Disorders of lipid metabolism (41.3)
	Arthritis (28.2)	Diabetes (28.8)	Diabetes (26.9)
	Varicose veins (26.9)	Substance abuse (24.3)	Obesity (19.7)
	Depression (24.7)	Obesity (17.6)	Arthritis (19.1)
	Diabetes (24.1)	Cancer (17.5)	Substance abuse (17.6)
	Obesity (23.4)	COPD ^5^ (14.6)	Depression (16.5)
	Osteoporosis (19.0)	Arthritis (13.7)	Cancer (16.0)
	Iron deficiency, anemia (17.4)	Cardiac arrhythmia (13.3)	Varicose veins (15.2)
	Hypothyroidism (16.3)	Iron deficiency, anemia (12.7)	Iron deficiency, anemia (14.4)

^1^ Standard error; ^2^ Defined as the presence of two or more chronic diseases, including chronic liver disease; ^3^ Standard deviation; ^4^ Expanded Diagnostic Clusters; ^5^ Chronic obstructive pulmonary disease.

**Table 2:** Prevalence of the most common chronic comorbidities in adult patients with chronic liver disease in the EpiChron Cohort stratified by sex and age.

Women Aged 18–39 Years (*n* = 142)			Men Aged 18–39 Years (*n* = 236)		
Mean Number of Chronic Diseases: 9.2			Mean Number of Chronic Diseases: 7.3		
EDC ^1^	Comorbidity	N (%)	EDC	Comorbidity	N (%)
CAR11	Dis. ^2^ of lipid metabolism	27 (19.0)	CAR11	Dis. of lipid metabolism	61 (25.7)
NUT03	Obesity	21 (14.8)	PSY02	Substance use	38 (16.0)
SKN02	Dermatitis and eczema	21 (14.8)	NUT03	Obesity	37 (15.6)
HEM02	Iron deficiency, anemia	20 (14.1)	ALL03	Allergic rhinitis	34 (14.3)
ALL03	Allergic rhinitis	18 (12.7)	SKN02	Dermatitis and eczema	30 (12.6)
END05	Other endocrine dis.	18 (12.7)	ASMA	Asthma	24 (10.1)
PSY01	Anxiety, neuroses	17 (12.0)	DIAB	Diabetes	20 (8.44)
SKN13	Disease of the hair	15 (10.6)	HTA	Arterial hypertension	18 (7.59)
END04	Hypothyroidism	14 (9.86)	MUS14	Low back pain	18 (7.59)
MUS14	Low back pain	14 (9.86)	PSY09	Depression	13 (5.48)
**Women Aged 40–64 Years (*n* = 1170)**			**Men Aged 40–64 Years (*n* = 2375)**		
**Mean Number of Chronic Diseases: 12.2**			**Mean Number of Chronic Diseases: 11.1**		
**EDC**	**Comorbidity**	**N (%)**	**EDC**	**Comorbidity**	**N (%)**
CAR11	Dis. of lipid metabolism	482 (41.2)	CAR11	Dis. of lipid metabolism	989 (41.6)
HTA	Arterial hypertension	366 (31.3)	HTA	Arterial hypertension	934 (39.3)
NUT03	Obesity	282 (24.1)	PSY02	Substance use	722 (30.4)
PSY09	Depression	280 (23.9)	DIAB	Diabetes	561 (23.6)
GSU08	Varicose veins	275 (23.5)	NUT03	Obesity	419 (17.6)
MUS03	Arthritis	208 (17.8)	PSY09	Depression	315 (13.3)
DIAB	Diabetes	190 (16.2)	CANCER	Cancer	274 (11.5)
END04	Hypothyroidism	188 (16.1)	SKN02	Dermatitis and eczema	259 (10.9)
PSY19	Sleep disorders	164 (14.0)	PSY19	Sleep disorders	258 (10.8)
END05	Other endocrine dis.	158 (13.5)	RES04	COPD ^3^	248 (10.4)
**Women Aged ≥ 65 Years (*n* = 1251)**			**Men Aged ≥ 65 Years (*n* = 1662)**		
**Mean Number of Chronic Diseases: 16.5**			**Mean Number of Chronic Diseases: 15.4**		
**EDC**	**Comorbidity**	**N (%)**	**EDC**	**Comorbidity**	**N (%)**
HTA	Arterial hypertension	908 (72.5)	HTA	Arterial hypertension	1059 (63.7)
CAR11	Dis. of lipid metabolism	574 (45.8)	CAR11	Dis. of lipid metabolism	714 (43.0)
MUS03	Arthritis	512 (40.9)	DIAB	Diabetes	649 (39.0)
DIAB	Diabetes	417 (33.3)	CANCER	Cancer	470 (28.3)
GSU08	Varicose veins	402 (32.1)	GUR04	Prostatic hypertrophy	414 (24.9)
END02	Osteoporosis	362 (28.9)	CAR09	Cardiac arrhythmia	405 (24.4)
PSY09	Depression	339 (27.1)	MUS03	Arthritis	374 (22.5)
NUT03	Obesity	294 (23.5)	RES04	COPD	374 (22.5)
HEM02	Iron deficiency, anemia	285 (22.8)	HEM02	Iron deficiency, anemia	326 (19.6)
EYE06	Cataract, aphakia	266 (21.2)	ADM02	Surgical aftercare	315 (19.0)

^1^ Expanded Diagnostic Clusters, ^2^ Disorder, ^3^ Chronic obstructive pulmonary disease.

**Table 3:** Chronic diseases systematically associated with the presence of chronic liver disease in the adult population of the EpiChron Cohort.

EDC ^1^	Comorbidity	Crude OR ^2^ (95% CI) ^3^	*p* Value	Adjusted OR ^4^ (95% CI)	*p* Value
GAS12	Chronic pancreatitis	66.1 (53.9–81.0)	<0.001	41.2 (33.5–50.6)	<0.00001
GTC02	Inherited metabolic disorders	22.9 (18.2–28.8)	<0.001	14.9 (11.8–18.8)	<0.00001
HEM07	Hemophilia, coagulation disorder	21.6 (17.0–27.5)	<0.001	14.0 (11.0–17.9)	<0.00001
HEM06	Deep-vein thrombosis	21.4 (16.8–27.3)	<0.001	13.4 (10.5–17.1)	<0.00001
INF04	HIV ^5^	14.4 (12.4–16.7)	<0.001	13.3 (11.4–15.4)	<0.00001
CAR10	Generalized atherosclerosis	20.1 (17.1–23.6)	<0.001	12.2 (10.3–14.4)	<0.00001
PSY13	Adjustment disorder	14.0 (10.4–18.9)	<0.001	11.0 (8.14–14.9)	<0.00001
PSY02	Substance use	13.9 (13.1–14.8)	<0.001	10.6 (9.93–11.3)	<0.00001
GAS08	Gastroesophageal reflux	15.1 (13.4–17.1)	<0.001	10.0 (8.85–11.3)	<0.00001
RHU01	Autoimmune, connective tissue dis.	12.5 (10.0–15.6)	<0.001	8.55 (6.84–10.7)	<0.00001
REN01	Chronic renal failure	10.8 (9.68–12.0)	<0.001	7.74 (6.91–8.67)	<0.00001
PSY20	Major depression	7.38 (5.14–10.6)	<0.001	7.01 (4.87–10.1)	<0.00001
CAR07	Cardiomyopathy	11.7 (9.46–14.5)	<0.001	6.66 (5.37–8.26)	<0.00001
RES13	Chronic respiratory failure	10.3 (8.09–13.1)	<0.001	6.40 (5.02–8.15)	<0.00001
RES06	Sleep apnea	10.3 (8.79–12.1)	<0.001	6.17 (5.25–7.25)	<0.00001
ADM02	Surgical aftercare	8.97 (8.34–9.66)	<0.001	6.02 (5.58–6.49)	<0.00001
REN06	End-stage renal disease	9.78 (6.80–14.1)	<0.001	5.67 (3.94–8.16)	<0.00001
RES11	Respiratory disorders, other	7.22 (6.65–7.84)	<0.001	4.55 (4.19–4.95)	<0.00001
ALL06	Disorders of the immune system	6.02 (5.22–6.94)	<0.001	4.36 (3.78–5.03)	<0.00001
NUR17	Paralytic syndromes, other	6.60 (5.13–8.48)	<0.001	4.10 (3.18–5.27)	<0.00001
CAR05	Congestive heart failure	5.24 (4.80–5.72)	<0.001	3.86 (3.52–4.25)	<0.00001
GSU15	Alimentary, excretory surgic. openings	6.44 (4.59–9.03)	<0.001	3.77 (2.69–5.30)	<0.00001
REN04	Nephritis, nephrosis	5.18 (4.09–6.56)	<0.001	3.74 (2.95–4.74)	<0.00001
RHU03	Arthropathy	5.74 (3.68–8.98)	<0.001	3.71 (2.37–5.80)	<0.00001
REN05	Renal disorders, other	5.08 (4.22–6.11)	<0.001	3.66 (3.04–4.40)	<0.00001
PSY08	Personality disorders	3.42 (2.81–4.16)	<0.001	3.34 (2.75–4.07)	<0.00001
CAR16	Cardiovascular disorders, other	5.06 (4.69–5.47)	<0.001	3.22 (2.98–3.48)	<0.00001
GAS10	Diverticular disease of the colon	4.72 (4.27–5.22)	<0.001	3.18 (2.87–3.52)	<0.00001
HEM02	Iron deficiency, anemias	3.68 (3.44–3.94)	<0.001	3.08 (2.87–3.30)	<0.00001
HEM08	Hematologic disorders, other	4.54 (4.20–4.90)	<0.001	2.93 (2.70–3.17)	<0.00001
CAR06	Cardiac valve disorders	4.42 (3.94–4.97)	<0.001	2.87 (2.55–3.24)	<0.00001
INF01	Tuberculosis infection	3.09 (2.56–3.73)	<0.001	2.83 (2.34–3.42)	<0.00001
DIAB	Diabetes	4.31 (4.08–4.55)	<0.001	2.72 (2.57–2.88)	<0.00001
EYE13	Diabetic retinopathy	4.24 (3.58–5.01)	<0.001	2.69 (2.27–3.18)	<0.00001
GSU11	Peripheral vascular disease	4.50 (3.80–5.32)	<0.001	2.62 (2.21–3.10)	<0.00001
RES04	COPD ^6^	4.34 (4.03–4.68)	<0.001	2.54 (2.34–2.74)	<0.00001
CAR09	Cardiac arrhythmia	3.75 (3.49–4.04)	<0.001	2.38 (2.21–2.57)	<0.00001
REC03	Chronic ulcer of the skin	3.37 (2.99–3.79)	<0.001	2.31 (2.04–2.61)	<0.00001
NUR07	Seizure disorder	2.64 (2.27–3.07)	<0.001	2.28 (1.96–2.65)	<0.00001
EYE03	Retinal disorders (excl. diabetic retinopathy)	3.20 (2.08–4.93)	<0.001	2.21 (1.43–3.41)	0.00033
CANCER	Cancer	3.26 (3.06–3.48)	<0.001	2.09 (1.96–2.24)	<0.00001
GUR09	Renal calculi	2.91 (2.58–3.29)	<0.001	1.99 (1.76–2.25)	<0.00001
NUT03	Obesity	2.62 (2.46–2.78)	<0.001	1.99 (1.87–2.11)	<0.00001
HEM01	Hemolytic anemia	1.82 (1.32–2.52)	0.00030	1.90 (1.37–2.63)	0.00012
NUR21	Neurologic disorders, other	2.47 (2.27–2.68)	<0.001	1.86 (1.71–2.03)	<0.00001
NUR03	Peripheral neuropathy, neuritis	2.14 (1.94–2.36)	<0.001	1.85 (1.68–2.04)	<0.00001
NUR19	Developmental disorder	0.85 (0.64–1.13)	0.272	1.85 (1.38–2.47)	0.00003
GAS02	Inflammatory bowel disease	2.25 (1.78–2.85)	<0.001	1.80 (1.42–2.28)	<0.00001
HTA	Hypertension	3.27 (3.11–3.43)	<0.001	1.79 (1.70–1.89)	<0.00001
RHU02	Gout	3.38 (3.05–3.75)	<0.001	1.77 (1.59–1.97)	<0.00001
RES08	Pulmonary embolism	2.71 (2.04–3.60)	<0.001	1.76 (1.32–2.33)	0.00010
GAS09	Irritable bowel syndrome	2.05 (1.77–2.37)	<0.001	1.69 (1.46–1.96)	<0.00001
MUS14	Low back pain	2.11 (1.95–2.29)	<0.001	1.68 (1.55–1.83)	<0.00001
HEM03	Thrombophlebitis	2.33 (2.06–2.64)	<0.001	1.66 (1.47–1.89)	<0.00001
PSY01	Anxiety, neuroses	1.73 (1.57–1.91)	<0.001	1.64 (1.48–1.82)	<0.00001
PSY09	Depression	2.02 (1.90–2.16)	<0.001	1.64 (1.53–1.75)	<0.00001
NUR24	Dementia	2.00 (1.73–2.32)	<0.001	1.58 (1.36–1.85)	<0.00001
IHD	Ischemic heart disease	2.74 (2.50–3.00)	<0.001	1.55 (1.41–1.70)	<0.00001
END04	Hypothyroidism	1.63 (1.50–1.77)	<0.001	1.54 (1.41–1.68)	<0.00001
END02	Osteoporosis	1.74 (1.60–1.89)	<0.001	1.50 (1.37–1.65)	<0.00001
END05	Other endocrine disorders	1.57 (1.44–1.72)	<0.001	1.50 (1.38–1.64)	<0.00001
PSY07	Schizophrenia, affective psychosis	1.90 (1.59–2.25)	<0.001	1.50 (1.26–1.78)	<0.00001
NUR05	Cerebrovascular disease	2.24 (2.02–2.48)	<0.001	1.39 (1.25–1.54)	<0.00001
MUS13	Cervical pain syndromes	1.67 (1.45–1.92)	<0.001	1.35 (1.17–1.56)	0.00003
PSY19	Sleep disorders	1.88 (1.75–2.02)	<0.001	1.35 (1.26–1.45)	<0.00001
SKN12	Psoriasis	1.71 (1.51–1.94)	<0.001	1.34 (1.18–1.52)	<0.00001
GSU08	Varicose veins of the lower extremities	1.58 (1.48–1.68)	<0.001	1.30 (1.21–1.40)	<0.00001
CAR11	Disorders of lipid metabolism	2.25 (2.14–2.36)	<0.001	1.27 (1.21–1.34)	<0.00001
SKN02	Dermatitis and eczema	0.89 (0.82–0.96)	0.00168	1.23 (1.14–1.33)	<0.00001
EYE06	Cataract, aphakia	1.84 (1.70–1.99)	<0.001	1.17 (1.07–1.27)	0.00029
MUS03	Arthritis	1.82 (1.71–1.93)	<0.001	1.17 (1.10–1.25)	<0.00001

^1^ Expanded Diagnostic Cluster; ^2^ Non-adjusted odds ratios; ^3^ 95% confidence interval; ^4^ Age- and sex-adjusted odds ratios; ^5^ Human immunodeficiency virus; ^6^ Chronic obstructive pulmonary disease. Only those comorbidities with a statistically significant age- and sex-adjusted odds ratio are presented in the table ordered from highest to lowest based on the value of the odds ratio.

## Data Availability

The data used in this study cannot be publicly shared because of restrictions imposed by the Aragon Health Sciences Institute (IACS) and asserted by the CEICA due to the existence of potentially identifying patient information. The authors who accessed the data received permission from IACS to utilize the data for this specific study, thus implying its exclusive use by the researchers appearing in the project protocol approved by the CEICA. Potential collaborations should be addressed to the Principal Investigator of the EpiChron Research Group, Antonio Gimeno-Miguel, at agimenomi.iacs@aragon.es. Requests for the data set used in this study should be addressed to CEICA at ceica@aragon.es.

## Supplementary Materials

The following supporting information can be downloaded at: www.mdpi.com/article/10.3390/jcm13237198/s1. Table S1: List of 110 chronic conditions or Expanded Diagnostic Clusters (EDC) included and analyzed in this study.
