# Supplementary material for: Multimorbidity in Patients with Chronic Liver Disease: A Population-Based Study in the EpiChron Cohort, Spain
Source: J Clin Med. 2024 Nov 27;13(23):7198. doi: 10.3390/jcm13237198 (PMC11642432; doi:10.3390/jcm13237198)
Supplement: Supplementary file 1 [file jcm-13-07198-s001.zip › jcm-3315208-supplementary.pdf]

Table S1. List of 110 chronic conditions or Expanded Diagnostic Clusters (EDC) included and analyzed in the study.

**EDC**	**Description**
ADM02	Surgical aftercare
ALL03	Allergic rhinitis
ASMA	Asthma
ALL06	Disorders of the immune system
IHD	Ischemic heart disease
CAR04	Congenital heart disease
CAR05	Congestive heart failure
CAR06	Cardiac valve disorders
CAR07	Cardiomyopathy
CAR09	Cardiac arrhythmia
CAR10	Generalized atherosclerosis
CAR11	Disorders of lipid metabolism
HTA	Arterial hypertension
CAR16	Cardiovascular disorders, other
EAR08	Deafness, hearing loss
END02	Osteoporosis
END04	Hypothyroidism
END05	Other endocrine disorders
DIAB	Diabetes
EYE02	Blindness
EYE03	Retinal disorders (excluding diabetic retinopathy)
EYE06	Cataract, aphakia
EYE08	Glaucoma
EYE13	Diabetic retinopathy
FRE03	Endometriosis
FRE12	Utero-vaginal prolapse
GAS02	Inflammatory bowel disease
GAS05	Chronic liver disease
GAS08	Gastroesophageal reflux
GAS09	Irritable bowel syndrome
GAS10	Diverticular disease of colon
GAS12	Chronic pancreatitis
GAS13	Lactose intolerance
GSU06	Chronic cystic disease of the breast
GSU08	Varicose veins of lower extremities
GSU11	Peripheral vascular disease
GSU13	Aortic aneurysm
GSU15	Alimentary or excretory surgical openings
GTC01	Chromosomal anomalies
GTC02	Inherited metabolic disorders
GUR01	Vesicoureteral reflux

GUR03	Hypospadias, other penile anomalies
GUR04	Prostatic hypertrophy
GUR09	Renal calculi
GUR10	Prostatitis
HEM01	Hemolytic anemia
HEM02	Iron deficiency, other deficiency anemias
HEM03	Thrombophlebitis
HEM05	Aplastic anemia
HEM06	Deep vein thrombosis
HEM07	Hemophilia, coagulation disorder
HEM08	Hematologic disorders, other
HEM09	Sickle cell disease
INF01	Tuberculosis infection
INF04	HIV, AIDS
CANCER	Cancer
ARTRITIS	Arthritis
MUS06	Kyphoscoliosis
MUS07	Congenital hip dislocation
MUS11	Congenital anomalies of limbs, hands, and feet
MUS13	Cervical pain syndromes
MUS14	Low back pain
NUR03	Peripheral neuropathy, neuritis
NUR05	Cerebrovascular disease
NUR06	Parkinson's disease
NUR07	Seizure disorder
NUR08	Multiple sclerosis
NUR09	Muscular dystrophy
NUR12	Quadriplegia and paraplegia
NUR16	Spinal cord injury/disorders
NUR17	Paralytic syndromes, other
NUR18	Cerebral palsy
NUR19	Developmental disorder
NUR21	Neurologic disorders, other
NUR24	Dementia
NUR26	Autism Spectrum Disorder
NUT03	Obesity
PSY01	Anxiety, neuroses
PSY02	Substance use
PSY05	Attention deficit disorder
PSY07	Schizophrenia and affective psychosis
PSY08	Personality disorders
PSY09	Depression
PSY13	Adjustment disorder
PSY14	Psychosocial disorders of childhood
PSY15	Eating disorder
PSY16	Impulse control

PSY17	Psycho-physiologic and somatoform disorders
PSY19	Sleep disorders of nonorganic origin
PSY20	Major depression
REC01	Cleft lip and palate
REC03	Chronic ulcer of the skin
REN01	Chronic renal failure
REN04	Nephritis, nephrosis
REN05	Renal disorders, other
REN06	End stage renal disease
RES03	Cystic fibrosis
RES04	Emphysema, chronic bronchitis, COPD
RES06	Sleep apnea
RES08	Pulmonary embolism
RES09	Tracheostomy
RES11	Respiratory disorders, other
RES13	Chronic respiratory failure
RHU01	Autoimmune and connective tissue diseases
RHU02	Gout
RHU03	Arthropathy
SKN02	Dermatitis and eczema
SKN12	Psoriasis
SKN13	Disease of hair and hair follicles
